# Carbonic anhydrase inhibition with a series of novel benzenesulfonamide-triazole conjugates

**DOI:** 10.1080/14756366.2018.1513927

**Published:** 2018-10-02

**Authors:** Marwa G. El-Gazzar, Nessma H. Nafie, Alessio Nocentini, Mostafa M. Ghorab, Helmi I. Heiba, Claudiu T. Supuran

**Affiliations:** a Department of Drug Radiation Research, National Center for Radiation Research and Technology (NCRRT), Egyptian Atomic Energy Authority (EAEA), Cairo, Egypt;; b NEUROFARBA Department, Pharmaceutical and Nutraceutical Sciences Section, University of Florence, Firenze, Italy

**Keywords:** Carbonic anhydrase, benzenesulfonamide, 1,2,3-triazole, hCA IX, anti-proliferative

## Abstract

We report the synthesis and characterisation of a novel series of triazole benzenesulfonamide derivatives, which incorporate the general pharmacophore associated with carbonic anhydrase (CA, EC 4.2.1.1) inhibitors. The synthesised compounds were tested *in vitro* against four human carbonic anhydrase (hCA, EC 4.2.1.1) isozymes, hCA I, hCA II, hCA IV and hCA IX. The obtained results showed that the tumour-associated hCA IX was the most sensitive to inhibition with the synthesised derivatives, with the triazolo-pyridine benzenesulfonamides **14**, **16** and **17** being the most effective inhibitors. Some selected compounds were chosen for a single dose anti-proliferative activity testing against a panel of 57 human tumour cell lines and show some anti-proliferative activity *ex vivo*.

## 1. Introduction

Carbonic anhydrases (CA, EC 4.2.1.1) are a large family of zinc-containing metallo-enzymes that catalyse the reversible hydration of carbon dioxide to hydrogen carbonate and H^+1,2^. In humans (h), 15 CA isoforms are known differing in tissue expression patterns, kinetic properties and subcellular localisation^3^. Their physiological roles are typically associated with acid–base homeostasis and the transport of CO_2_ and hydrogen carbonate. Human (h) isoform hCA IX is a transmembrane protein with an extracellular active site, and is poorly expressed in healthy tissues (as GIT, bile duct and gall bladder), being instead over-expressed in many solid tumours as a result of hypoxia^4^^,^^5^. The function of hCA IX in tumour cell is to maintain acid–base homeostasis under hypoxic conditions and to facilitate the diffusion of H^+^ through the entire solid tumour leading to low extracellular pH that produces matrix breakdown, invasion, immune suppression and multi-drug resistance leading to more tumour aggression and resistance^6^^,^^7^. These findings led to great interest for new therapeutics targeting hCA IX. Inhibition of hCA IX with small molecules has emerged as a novel anticancer strategy^8–10^. The most important and widely studied class of CA inhibitors are the aromatic sulfonamides which are capable to coordinate the catalytic Zn^2+^ from the enzyme active site, thus blocking the catalytic process^11–16^. Moreover, several 1,2,3-triazole containing compounds have proved considerable biological activities including antibacterial, antifungal and anticancer activities^17–22^.

**Figure 1.**
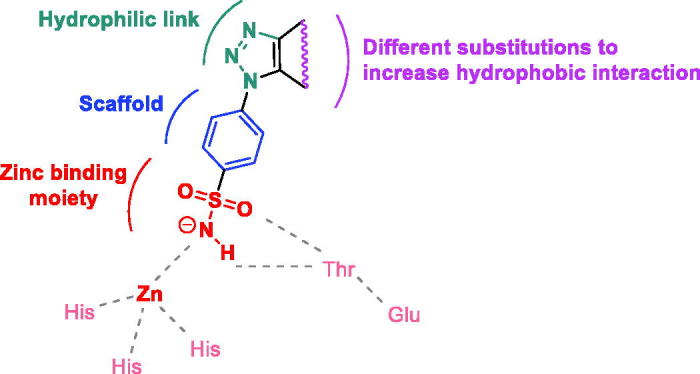
Design of the synthesised compounds.

In view of these facts, and in continuation of an ongoing project aiming to develop new biologically active sulfonamide derivatives^23–30^, we report herein a new set of triazole-benzenesulfonamides designed in agreement with the general pharmacophoric requirements for hCA IX inhibition: an aromatic sulfonamide moiety is used as base unit for the synthesis of the target compounds since necessary to coordinate with the Zn atom and bind to pivotal amino-acids in the active site pocket. An 1,2,3-triazole ring is appended at the aromatic scaffold and used as hydrophilic linker to incorporate several substitution patterns planned to increase the hydrophobic interactions within the active site cavity (Figure 1).

The synthesised compounds were tested for their inhibitory activity assay against four CA isoforms (hCA I, hCA II, hCA IV and hCA IX). Moreover, they were further evaluated against a panel of 57 human cell lines at National Cancer Institute (NCI, Bethesda, MD).

## 2. Materials and methods

### 2.1. Instruments

Melting points were taken in an open capillary tube on a Stuart melting point apparatus (Stuart Scientific, Redhill, UK) and were uncorrected. The IR spectra of the compounds were recorded on FTIR Shimadzu spectrometer (Shimadzu, Tokyo, Japan). ^1^H NMR and 13C NMR spectra were recorded on a Varian Mercury Plus Oxford (300 MHz for ^1^H-NMR and 75 MHz for 13C-NMR) spectrometer (Varian Inc., Palo Alto, CA) using TMS as an internal Standard and DMSO-d_6_ as solvent. Mass spectra were run on HP Model MS-5988 (Hewlett Packard, Palo, Alto, CA). Microanalyses were obtained on a Carlo Erba 1108 Elemental Analyzer (Heraeus, Hanau, Germany). All values were within ±0.4% of the theoretical values. Purity of the compounds was checked by TLC on pre-coated SiO_2_ gel (HF254, 200 mesh) aluminium plates (Merk, Darmstadt, Germany). A developing solvent system of chloroform/methanol (8:2) was used and the spots were visualised under UV light. IR, ^1^H NMR, 13C NMR, Mass and elemental analysis were consistent with the assigned structures. Starting sulfanilamide and all reagents used were of analytical grade and were purchased from Sigma (St. Louis, MO).

### 2.2. Chemistry

#### 4-(5-amino-4-cyano-1H-1,2,3-triazol-1-yl)benzenesulfonamide 3

A mixture of **2** (1 g, 0.005 mol) and malononitrile (0.33 g, 0.005 mol) was stirred in ethanol containing sodium ethoxide (0.11 g, 0.005 mol) at room temperature overnight and the precipitated solid was filtered off and crystallised from acetic acid to give **3**. Yield = 85%; m.p.:100–101 °C. IR(cm^−1^): 3335, 3205 (NH_2_), 3096 (CH arom.), 2231 (CN), 1315, 1163 (SO_2_). ^1^H-NMR (300 MHz, DMSO-d_6_): *δ* 7.59 (d, 2H, Ar-H, *J* = 8.8 Hz), 7.63 (s, 2H, NH_2_, D_2_O exch.), 7.89 (d, 2H, Ar-H, *J* = 8.8 Hz), 8.5 (s, 2H, NH_2_, D_2_O exch.). 13C-NMR (75 MHz, DMSO-d_6_): *δ* 113.97, 118.1 (2), 128.55(2), 137.48, 138.24, 139.36, 150.62. MS, *m/z* (%): 264(M^+^). Anal. Calcd. For C_9_H_8_N_6_O_2_S (264): C, 40.91; H, 3.05; N, 31.80; Found: C, 40.69; H, 3.35; N, 31.65.

#### 4-(5-amino-4-(4,5-dihydro-1H-imidazol-2-yl)-1H-1,2,3-triazol-1-yl)benzenesulfonamide 4

A mixture of **3** (0.3 g, 0.001 mol) in ethylenediamine (7 ml) and carbon disulfide (7 ml) was heated under reflux for 6 h. After cooling, the reaction mixture was poured onto cold water and the formed solid was filtered off and crystallised from ethanol to give **4**. Yield = 80%; m.p.: 278–280 °C. IR(cm^−1^): 3372, 3183 (NH_2_), 3002 (CH arom.), 1610 (C = N), 1312, 1116 (SO_2_). ^1^H-NMR (300 MHz, DMSO-d_6_): *δ* 3.33 (d, 2H, CH imidazole, *J* = 13.0 Hz), 3.61 (d, 2H, CH imidazole, *J* = 13.2 Hz), 7.49 (d, 2H, Ar-H, *J* = 8.8 Hz), 7.65 (s, 2H, NH_2_, D_2_O exch.) 7.86 (d, 2H, Ar-H, *J* = 8.8 Hz), 8.2 (s, 2H, NH_2_, D_2_O exch.). 13C-NMR (75 MHz, DMSO-d_6_): *δ* 49.41 (2), 118.10 (2), 128.55 (2), 137.48, 139.36, 138.24, 150.62, 152.89. MS, *m/z* (%): 307(M^+^). Anal. Calcd. For C_11_H_13_N_7_O_2_S (307): C, 42.99; H, 4.26; N, 31.90; Found: C, 42.75; H, 4.34; N, 31.77.

#### 2-chloro-*N*-(4-cyano-1-(4-sulfamoylphenyl)-1H-1,2,3-triazol-5-yl)acetamide 5

A mixture of **3** (0.3 g, 0.001 mol) and chloroacetyl chloride (0.15 g, 0.001 mol) was stirred in DMF for 2 h, the reaction mixture was poured onto cold water and the formed solid was filtered off and crystallised from ethanol to give **5**. Yield = 91%; m.p.: 165–167 °C. IR(cm^−1^): 3337, 3187 (NH, NH_2_), 3099 (CH arom.), 2933, 2858 (CH aliph.), 2231 (CN), 1668 (C = O), 1345, 1112 (SO_2_). ^1^H-NMR (300 MHz, DMSO-d_6_): *δ* 4.26 (s, 2H, CH_2_Cl), 7.52 (d, 2H, Ar-H, *J* = 8.6 Hz), 7.69 (s, 2H, NH_2_, D_2_O exch.), 7.90 (d, 2H, Ar-H, *J* = 8.6 Hz), 9.5 (s, 1H, NH, D_2_O exch.). 13C-NMR (75 MHz, DMSO-d_6_): *δ* 42.64, 113.97, 118.10 (2), 128.55 (2), 137.48, 138.24, 139.36, 150.62, 164.79. MS, *m/z* (%): 340(M^+^). Anal. Calcd. For C_11_H_9_ClN_6_O_3_S (340): C, 38.77; H, 2.66; N, 24.66; Found: C, 38.85; H, 2.46; N, 24.45.

#### *N*-(4-cyano-1-(4-sulfamoylphenyl)-1H-1,2,3-triazol-5-yl)-3-oxobutanamide 6

A mixture of **3** (0.3 g, 0.001 mol) and ethyl acetoacetate (0.13 g, 0.001 mol) was refluxed in ethanol for 5 h, the reaction mixture was cooled and the formed solid was filtered off and crystallised from ethanol to give **6**. Yield = 95%; m.p.: >300 °C. IR(cm^−1^): 3342, 3270 (NH, NH_2_), 3097 (CH arom.), 2970, 2890 (CH aliph.), 2205 (CN), 1707 (2C = O), 1342, 1125 (SO_2_). ^1^H-NMR (300 MHz, DMSO-d_6_): *δ* 2.13 (s, 3H, CH_3_), 3.58 (s, 2H, CH_2_), 7.69 (s, 2H, NH_2_, D_2_O exch.) 7.52 (d, 2H, Ar-H, *J* = 8.6 Hz), 7.90 (d, 2H, Ar-H), *J* = 8.6 Hz), 9.1 (s, 1H, NH, D_2_O exch.). 13C-NMR (75 MHz, DMSO-d_6_): *δ* 30.82, 50.25, 113.97, 118.1 (2), 128.55 (2), 137.48, 138.24, 139.36, 150.62, 170.59, 204.29. MS, *m/z* (%): 348 (M^+^). Anal. Calcd. For C_13_H_12_N_6_O_4_S (348): C, 44.83; H, 3.47; N, 24.13; Found: C, 44.90; H, 3.51; N, 24.35.

#### 4-(4-cyano-5-((2-oxo-2-phenylethyl)amino)-1H-1,2,3-triazol-1-yl)benzenesulfonamide 7

A mixture of **3** (0.3 g, 0.001 mol) and phenacyl bromide (0.2 g, 0.001 mol) was refluxed in ethanol for 3 h, the reaction mixture was cooled, poured onto ice water and the formed solid was filtered off and crystallised from acetone to give **7**. Yield = 89%; m.p. 216–218 °C. IR(cm^−1^): 3339, 3206 (NH, NH_2_), 3088 (CH arom.), 2925, 2889 (CH aliph.), 2224 (CN), 1677 (C = O), 1343, 1119 (SO_2_). ^1^H-NMR (300 MHz, DMSO-d_6_): *δ* 4.03 (s, 2H, CH_2_), 7.48–7.58 (m, 5H, Ar-H), 7.59 (d, 2H, Ar-H, *J* = 8.7 Hz), 7.66 (d, 2H, Ar-H, *J* = 8.8 Hz), 7.88 (s, 2H, NH_2_, D_2_O exch.), 10.1 (s, 1H, NH, D_2_O exch.). 13C-NMR (75 MHz, DMSO-d_6_): *δ* 50.35, 113.97, 118.10 (2), 128.47 (2), 128.55 (2), 128.89 (2), 128.92, 133.80, 137.48, 138.24, 139.36, 150.62, 191.85. MS, *m/z* (%): 382 (M^+^). Anal. Calcd. For C_17_H_14_N_6_O_3_S (382): C, 53.40; H, 3.69; N, 21.98; Found: C, 53.72; H, 3.61; N, 21.77.

#### General procedure for the synthesis of compounds 8–10

##### (E)-4-(5-((substituted-benzylidene)amino)-4-cyano-1H-1,2,3-triazol-1-yl)-benzenesulfonamide 8–10.

A mixture of **3** (0.3 g, 0.001 mol) and the appropriate aromatic aldehyde (0.001 mol) was refluxed in acetic acid for 5 h and the precipitate formed while hot was filtered off and crystallised from ethanol to give **8–10**, respectively.

##### (E)-4-(5-((2-chlorobenzylidene)amino)-4-cyano-1H-1,2,3-triazol-1-yl)-benzenesulfonamide 8

Yield = 76%; m.p(0).210–211 °C. IR(cm^−1^): 3342, 3245 (NH_2_), 3088 (CH arom.), 2188 (CN), 1334, 1120 (SO_2_), 657 (C-Cl). ^1^H-NMR (300 MHz, DMSO-d_6_): *δ* 7.38–7.60 (m, 4H, Ar-H), 7.72 (s, 2H, NH_2_, D_2_O exch.), 7.96 (d, 2H, Ar-H, *J* = 8.4 Hz), 7.99 (d, 2H, Ar-H, *J* = 8.5 Hz)), 8.87 (s, 1H, CH). 13C-NMR (75 MHz, DMSO-d_6_): *δ* 113.97, 118.1 (2), 127.10, 128.70, 128.55 (2), 130.00, 130.55, 133.39, 136.10, 137.48, 138.24, 139.36, 151.50, 159.86. MS, *m/z* (%): 386 (M^+^). Anal. Calcd. For C_16_H_11_ClN_6_O_2_S (386): C, 49.68; H, 2.87; N, 21.73; Found: C, 49.81; H, 3.01; N, 21.82.

##### (E)-4-(5-((4-chlorobenzylidene)amino)-4-cyano-1H-1,2,3-triazol-1-yl) benzenesulfonamide 9

Yield = 80%; m.p.: 270–272 °C. IR(cm^−1^): 3335, 3213 (NH_2_), 3087 (CH arom.), 2197 (CN), 1343, 1123 (SO_2_), 659 (C-Cl). ^1^H-NMR (300 MHz, DMSO-d_6_): *δ* 7.49 (d, 2H, Ar-H, *J* = 8.4 Hz), 7.55 (d, 2H, Ar-H, *J* = 8.4 Hz), 7.97 (d, 2H, Ar-H, *J* = 8.4 Hz), 7.72 (s, 2H, NH_2_, D_2_O exch.), 7.96 (d, 2H, Ar-H, *J* = 8.4 Hz), 8.86 (s, 1H, CH). 13C-NMR (75 MHz, DMSO-d_6_): *δ* 118.1 (2), 113.97, 128.55 (2), 129.26 (2), 129.45 (2), 134.64, 135.68, 137.48, 138.24, 139.36, 151.50, 159.86. MS, *m/z* (%): 386 (M^+^). Anal. Calcd. For C_16_H_11_ClN_6_O_2_S (386): C, 49.68; H, 2.87; N, 21.73; Found: C, 48.77; H, 2.99; N, 21.81.

##### (E)-4-(4-cyano-5-((4-(dimethylamino)benzylidene)amino)-1H-1,2,3-triazol-1-yl)benzenesulfonamide 10

Yield = 89%; m.p.: 200–201 °C. IR(cm^−1^): 3336, 3233 (NH_2_), 3090 (CH arom.), 2207 (CN), 1333, 1115 (SO_2_). ^1^H-NMR (300 MHz, DMSO-d_6_): *δ* 2.92 (s, 6H, 2CH_3_), 6.72 (d, 2H, Ar-H, *J* = 8.4 Hz), 7.72 (d, 2H, Ar-H, *J* = 8.4 Hz), 7.70 (s, 2H, NH_2_, D_2_O exch.), 7.91 (d, 2H, Ar-H, *J* = 8.4 Hz), 7.92 (d, 2H, Ar-H, *J* = 8.4 Hz), 9.12 (s, 1H, CH). 13C-NMR (75 MHz, DMSO-d_6_): *δ* 40.30 (2), 111.54 (2), 113.97, 118.10 (2), 128.55 (2), 130.30 (2), 134.64, 137.48, 138.24, 139.36, 151.43, 151.50, 159.86. MS, *m/z* (%): 395 (M^+^). Anal. Calcd. For C_18_H_17_N_7_O_2_S (395): C, 54.67; H, 4.33; N, 24.79; Found: C, 54.81; H, 4.45; N, 24.59.

#### General procedure for the synthesis of compounds 11and 10

A mixture of **3** (0.3 g, 0.001 mol) and benzene or toluene sulfonyl chloride (0.001 mol) was refluxed in pyridine for 8 h, the reaction mixture was cooled, poured onto ice water and the formed solid was filtered off and crystallised from ethanol to give **11** and **12**, respectively.

##### *N*-(4-cyano-1-(4-sulfamoylphenyl)-1H-1,2,3-triazol-5-yl)benzenesulfonamide 11

Yield = 78%; m.p.: >300 °C. IR(cm^−1^): 3319, 3231 (NH, NH_2_), 3089 (CH arom.), 2210 (CN), 1321, 1124 (SO_2_). ^1^H-NMR (300 MHz, DMSO-d_6_): *δ* 7.44 (d, 2H, Ar-H, *J* = 8.5 Hz), 7.49–7.68 (m, 5H, Ar-H), 7.74 (d, 2H, Ar-H, *J* = 8.5 Hz), 7.90 (s, 2H, NH_2_, D_2_O exch.), 8.21 (s, 1H, NH, D_2_O exch.). 13C-NMR (75 MHz, DMSO-d_6_): *δ* 113.97, 118.10 (2), 127.00 (2), 128.55 (2), 129.20 (2), 131.44, 135.14, 137.48, 138.24, 139.36, 150.62. MS, *m/z* (%): 404 (M^+^). Anal. Calcd. For C_15_H_12_N_6_O_4_S_2_ (404): C, 44.55; H, 2.99; N, 20.78; Found: C, 44.77; H, 2.78; N, 20.58.

##### *N*-(4-cyano-1-(4-sulfamoylphenyl)-1H-1,2,3-triazol-5-yl)-4-methylbenzenesulfonamide 12

Yield = 89%; m.p.: >300 °C. IR(cm^−1^): 3338, 3231 (NH, NH_2_), 3066 (CH arom.), 2928, 2843 (CH aliph.), 2224 (CN), 1333, 1114 (SO_2_). ^1^H-NMR (300 MHz, DMSO-d_6_): *δ* 2.33 (s, 3H, CH_3_), 7.32 (d, 2H, Ar-H, *J* = 8.1 Hz), 7.44 (d, 2H, Ar-H, *J* = 8.5 Hz), 7.62 (d, 2H, Ar-H, *J* = 8.1 Hz), 7.90 (d, 2H, Ar-H, *J* = 8.7 Hz). 7.94 (s, 2H, NH_2_, D_2_O exch.), 8.27 (s, 1H, NH, D_2_O exch.). 13C-NMR (75 MHz, DMSO-d_6_): *δ* 21.26, 113.97, 118.10 (2), 127.50 (2), 128.55 (2), 129.69 (2), 135.14, 137.48, 138.24, 139.36, 144.26, 150.62. MS, *m/z* (%): 418 (M^+^). Anal. Calcd. For C_16_H_14_N_6_O_4_S_2_ (418): C, 45.93; H, 3.37; N, 20.08; Found: C, 45.68; H, 2.91; N, 20.48.

##### 4-(7-amino-6-cyano-5-oxo-4,5-dihydro-3H-[1,2,3]triazolo[4,5-b]pyridin-3-yl) benzenesulfonamide 13

Fusion of **3** (0.3 g, 0.001 mol) with ethyl cyanoacetate (0.1 g, 0.001 mol) was done for 10 min, the reaction mixture was cooled, triturated with diethyl ether and the formed solid was filtered off and crystallised from ethanol to give **13**. Yield = 78%; m.p.: >300 °C. IR(cm^−1^): 3339–3195 (NH, 2NH_2_), 3080 (CH arom.), 2210 (CN), 1698 (C = O), 1323, 1141 (SO_2_). ^1^H-NMR (300 MHz, DMSO-d_6_): *δ* 7.55 (d, 2H, Ar-H, *J* = 8.5 Hz), 7.71 (s, 2H, NH_2_, D_2_O exch.), 7.89 (d, 2H, Ar-H, *J* = 8.5 Hz), 8.02 (s, 2H, NH_2_, D_2_O exch.), 8.89 (s, 1H, NH, D_2_O exch.). 13C-NMR (75 MHz, DMSO-d_6_): *δ* 91.52, 115.02, 118.10 (2), 128.55 (2), 137.48, 138.24, 139.36, 150.62, 163.08, 163.15. MS, *m/z* (%): 331 (M^+^). Anal. Calcd. For C_12_H_9_N_7_O_3_S (331): C, 43.50; H, 2.74; N, 29.59; Found: C, 43.71; H, 2.55; N, 29.38.

#### General procedure for the synthesis of compounds 14–17

##### 4-(7-amino-6-cyano-5-(substituted)-3H-[1,2,3]triazolo[4,5-b]pyridin-3-yl)-benzenesulfonamide 14–17.

A mixture of **3** (0.3 g, 0.001 mol) and the appropriate benzylidene derivative (0.001 mol) was refluxed in ethanol containing catalytic amount of TEA (0.01 ml) for 5 h, the reaction mixture was left to cool and the precipitate formed was filtered off and crystallised from dioxane to give **14–17**, respectively.

##### 4-(7-amino-6-cyano-5-phenyl-3H-[1,2,3]triazolo[4,5-b]pyridin-3-yl)benzenesulfonamide 14

Yield = 70%; m.p.: >300 °C. IR(cm^−1^): 3356–3252 (2NH_2_), 3077 (CH arom.), 2192 (CN), 1313, 1125 (SO_2_). ^1^H-NMR (300 MHz, DMSO-d_6_): *δ* 7.51 (s, 2H, NH_2_, D_2_O exch.), 7.70–7.80 (m, 5H, Ar-H), 7.87 (d, 2H, Ar-H, *J* = 7.1 Hz), 7.96 (d, 2H, Ar-H, *J* = 7.1 Hz), 8.35 (s, 2H, NH_2_, D_2_O exch.). 13C-NMR (75 MHz, DMSO-d_6_): *δ* 116.59, 118.10 (2), 128.55 (2), 128.86 (2), 128.87 (2), 128.92, 130.66, 136.57, 138.24, 139.36, 140.47, 141.02, 156.05, 159.95. MS, *m/z* (%): 391 (M^+^). Anal. Calcd. For C_18_H_13_N_7_O_2_S (391): C, 55.24; H, 3.35; N, 25.05; Found: C, 55.56; H, 3.15; N, 25.29.

##### 4-(7-amino-5-(2-chlorophenyl)-6-cyano-3H-[1,2,3]triazolo[4,5-b]pyridin-3-yl)benzenesulfonamide 15

Yield = 87%; m.p.: 115–117 °C. IR (cm^−1^): 3330–3223 (2NH_2_), 3087 (CH arom.), 2195 (CN), 1333, 1121 (SO_2_), 678 (C-Cl). ^1^H-NMR (300 MHz, DMSO-d_6_): *δ* 7.51 (s, 2H, NH_2_, D_2_O exch.), 7.59–7.77 (m, 4H, Ar-H), 7.89 (d, 2H, Ar-H, *J* = 8.4 Hz), 8.05 (d, 2H, Ar-H, *J* = 8.4 Hz), 8.08 (s, 2H, NH_2_, D_2_O exch.). 13C-NMR (75 MHz, DMSO-d_6_): *δ* 116.59, 118.10 (2), 127.58, 127.82, 128.55 (2), 128.71, 130.55, 130.66 (2), 133.16, 138.24, 139.36, 140.47, 141.02, 156.05, 159.95. MS, *m/z* (%): 425 (M^+^). Anal. Calcd. For C_18_H_12_ClN_7_O_2_S (425): C, 50.77; H, 2.84; N, 23.02; Found: C, 50.56; H, 3.02; N, 23.22.

##### 4-(7-amino-5-(4-chlorophenyl)-6-cyano-3H-[1,2,3]triazolo[4,5-b]pyridin-3-yl)benzenesulfonamide 16

Yield = 87%; m.p.: >300 °C. IR(cm^−1^): 3358–3254 (2NH_2_), 3011 (CH arom.), 2191 (CN), 1333, 1121 (SO_2_), 688 (C-Cl). ^1^H-NMR (300 MHz, DMSO-d_6_): *δ* 7.12 (s, 2H, NH_2_, D_2_O exch.), 7.49 (d, 2H, Ar-H, *J* = 8.6 Hz), 7.89 (d, 2H, Ar-H, *J* = 8.4 Hz), 8.11 (d, 2H, Ar-H, *J* = 8.4 Hz), 8.21 (d, 2H, Ar-H, *J* = 8.6 Hz), 8.55 (s, 2H, NH_2_, D_2_O exch.). 13C-NMR (75 MHz, DMSO-d_6_): *δ* 116.59, 118.10 (2), 128.20 (2), 128.55 (2), 130.66, 130.70 (2), 135.68, 136.57, 138.24, 139.36. 140.47, 141.02, 156.05, 159.95. MS, *m/z* (%): 425 (M^+^). Anal. Calcd. For C_18_H_12_ClN_7_O_2_S (425): C, 50.77; H, 2.84; N, 23.02; Found: C, 50.58; H, 3.11; N, 23.29.

##### 4-(7-amino-6-cyano-5-(4-(dimethylamino)phenyl)-3H-[1,2,3]triazolo[4,5-b]pyridin-3-yl)benzenesulfonamide 17

Yield = 89%; m.p.: 130–132 °C. IR(cm^−1^): 3343–3211 (2NH_2_), 3081 (CH arom.), 2199 (CN), 1333, 1126 (SO_2_). ^1^H-NMR (300 MHz, DMSO-d_6_): *δ* 3.02 (s, 6H, 2CH_3_), 6.88 (s, 2H, NH_2_, D_2_O exch.), 7.37 (d, 2H, Ar-H, *J* = 8.4 Hz), 7.79 (d, 2H, Ar-H, *J* = 8.4 Hz), 7.82 (d, 2H, Ar-H, *J* = 8.8 Hz), 8.06 (d, 2H, Ar-H, *J* = 8.8 Hz), 8.50 (s, 2H, NH_2_, D_2_O exch.). 13C-NMR (75 MHz, DMSO-d_6_): *δ* 40.30 (2), 113.84 (2), 116.59, 118.10 (2), 127.82 (2), 128.55 (2), 130.66, 136.57, 138.24, 139.36, 140.47, 141.02, 151.43, 156.05, 159.95. MS, *m/z* (%): 434 (M^+^). Anal. Calcd. For C_20_H_18_N_8_O_2_S (434): C, 55.29; H, 4.18; N, 25.79; Found: C, 55.52; H, 3.89; N, 25.55.

##### 4-(7-amino-5-thioxo-4,5-dihydro-3H-[1,2,3]triazolo[4,5-d]pyrimidin-3-yl)benzenesulfonamide 18

Fusion of **3** (0.3 g, 0.001 mol) with thiourea (0.1 g, 0.001 mol) was done for 10 min, the reaction mixture was cooled, triturated with diethyl ether and the formed solid was filtered off and crystallised from ethanol to give **18**.

Yield = 91%; m.p. 285–287 °C. IR(cm^−1^): 3360–3198 (NH, 2NH_2_), 3089 (CH arom.), 1623 (C = S), 1323, 1121 (SO_2_). ^1^H-NMR (300 MHz, DMSO-d_6_): *δ* 7.18 (s, 2H, NH_2_, D_2_O exch.), 7.52 (d, 2H, Ar-H, *J* = 8.5 Hz), 7.90 (d, 2H, Ar-H, *J* = 8.5 Hz), 8.01 (s, 2H, NH_2_, D_2_O exch.), 8.55 (s, 1H, NH, D_2_O exch.). 13C-NMR (75 MHz, DMSO-d_6_): *δ* 118.10 (2), 128.55 (2), 137.48, 138.24, 139.36, 150.62, 152.89, 179.08. MS, *m/z* (%): 323 (M^+^). Anal. Calcd. For C_10_H_9_N_7_O_2_S_2_ (323): C, 37.15; H, 2.81; N, 30.32; Found: C, 37.43; H, 3.03; N, 30.10.

##### 4-(7-imino-5-oxo-6-phenyl-4,5,6,7-tetrahydro-3H-[1,2,3]triazolo[4,5-d]pyrimidin-3-yl)benzenesulfonamide 19

A mixture of **3** (0.3 g, 0.001 mol) and phenyl isocyanate (0.13 g, 0.001 mol) was refluxed in DMF containing catalytic amount of TEA (0.01 ml) for 18 h, the reaction mixture was cooled, poured onto ice water and the formed solid was filtered off and crystallised from ethanol to give **19**. Yield = 89%; m.p.: 180–182 °C. IR(cm^−1^): 3320, 3292, 3198, 3133 (2NH, NH_2_), 3064 (CH arom.), 1675 (C = O), 1343, 1123 (SO_2_). ^1^H-NMR (300 MHz, DMSO-d_6_): *δ* 7.47–7.55 (m, 5H, Ar-H), 7.52 (d, 2H, Ar-H, *J* = 8.8 Hz), 7.62 (s, 2H, NH_2_, D_2_O exch.), 7.89 (d, 2H, Ar-H, *J* = 8.8 Hz), 8.01, 8.11 (2 s, 2H, 2NH, D_2_O exch.). 13C-NMR (75 MHz, DMSO-d_6_): *δ* 118.10 (2), 122.11 (2), 124.72, 128.55 (2), 129.25 (2), 137.48, 138.24, 139.36, 144.73, 149.00, 150.62, 151.73. MS, *m/z* (%): 383 (M^+^). Anal. Calcd. For C_16_H_13_N_7_O_3_S (383): C, 50.13; H, 3.42; N, 25.57; Found: C, 50.37; H, 3.21; N, 25.38.

##### 4-(6-ethyl-7-imino-5-thioxo-4,5,6,7-tetrahydro-3H-[1,2,3]triazolo[4,5-d]pyrimidin-3-yl)benzenesulfonamide 20

A mixture of **3** (0.3 g, 0.001 mol) and ethyl isothiocyanate (0.13 g, 0.001 mol) was refluxed in pyridine for 10 h, the reaction mixture was cooled, poured onto ice water, acidified with diluted HCl and the formed solid was filtered off and crystallised from ethanol to give **20**. Yield = 90%; m.p. 278–279 °C. IR(cm^−1^): 3340–3214 (2NH, NH_2_), 3069 (CH arom.), 2929, 2843 (CH aliph.), 1601 (C = S), 1333, 1120 (SO_2_). ^1^H-NMR (300 MHz, DMSO-d_6_): *δ* 1.25 (t, 3H, CH_3_, *J* = 7.1 Hz), 3.85 (q, 2H, CH_2_, *J* = 7.1 Hz), 7.51 (d, 2H, Ar-H, *J* = 8.8 Hz), 7.89 (d, 2H, Ar-H, *J* = 8.8 Hz), 7.92 (s, 2H, NH_2_, D_2_O exch.), 8.00, 8.10 (2 s, 2H, 2NH, D_2_O exch.). 13C-NMR (75 MHz, DMSO-d_6_): *δ* 12.98, 44.37, 118.10 (2), 128.55 (2), 137.48, 138.24, 139.36, 149.00, 150.62, 177.16. MS, *m/z* (%): 351 (M^+^). Anal. Calcd. For C_12_H_13_N_7_O_2_S_2_ (351): C, 41.02; H, 3.73; N, 27.90; Found: C, 41.31; H, 3.51; N, 27.73.

##### 7-imino-*N*-(quinoxalin-2-yl)-3-(4-sulfamoylphenyl)-5-thioxo-3,4,5,7-tetrahydro-6H-[1,2,3]triazolo[4,5-d]pyrimidine-6-sulfonamide 21

A mixture of **3** (0.3 g, 0.001 mol) and 4-isothiocyanato-*N*-(quinoxalin-2-yl) benzenesulfonamide29^,^30 (0.4 g, 0.001 mol) was refluxed in DMF containing catalytic amount of TEA (0.01 ml) for 6 h, the reaction mixture was cooled, poured onto ice water and the formed solid was filtered off and crystallised from ethanol to give **21**. Yield = 89%; m.p.: 212–215 °C. IR(cm^−1^): 3435–3238 (3NH, NH_2_), 3074 (CH arom.), 2924, 2846 (CH aliph.), 1599 (C = S), 1320, 1146 (SO_2_). ^1^H-NMR (300 MHz, DMSO-d_6_): *δ* 7.51 (d, 2H, Ar-H, *J* = 8.7 Hz), 7.68–7.81 (m, 4H, Ar-H), 7.73 (d, 2H, Ar-H, *J* = 8.8 Hz), 7.91 (d, 2H, Ar-H, *J* = 7.9 Hz), 8.09 (s, 1H, CH quinoxaline), 8.10 (d, 2H, Ar-H, *J* = 8.0 Hz), 8.22 (s, 2H, NH_2_, D_2_O exch.), 8.32, 8.36, 8.55 (3 s, 3H, 3NH, D_2_O exch.). 13C-NMR (75 MHz, DMSO-d_6_): *δ* 117.29 (2), 118.10 (2), 126.14 (2), 128.08 (2), 128.55 (3), 128.71, 129.18, 133.28, 136.75, 137.48, 138.24, 139.36, 143.64, 144.73, 149.00, 150.62, 153.92, 177.16. MS, *m/z* (%): 530 (M^+^). Anal. Calcd. For C_24_H_18_N_10_O_4_S_2_ (606): C, 47.52; H, 2.99; N, 23.09; Found: C, 47.41; H, 2.72; N, 23.25.

### 2.3. Carbonic anhydrase inhibition

An Applied Photophysics stopped-flow instrument has been used for assaying the CA-catalysed CO_2_ hydration activity^31^. Phenol red (at a concentration of 0.2 mM) has been used as indicator, working at the absorbance maximum of 557 nm, with 20 mM Hepes (pH 7.5) as buffer, and 20 mM Na_2_SO_4_ (for maintaining constant the ionic strength), following the initial rates of the CA-catalysed CO_2_ hydration reaction for a period of 10–100 s. The CO_2_ concentrations ranged from 1.7 to 17 mM for the determination of the kinetic parameters and inhibition constants. For each inhibitor at least six traces of the initial 5–10% of the reaction have been used for determining the initial velocity. The uncatalysed rates were determined in the same manner and subtracted from the total observed rates. Stock solutions of inhibitor (0.1 mM) were prepared in distilled-deionised water and dilutions up to 0.01 nM were done thereafter with the assay buffer. Inhibitor and enzyme solutions were pre-incubated together for 15 min at room temperature prior to assay, in order to allow for the formation of the E-I complex. The inhibition constants were obtained by non-linear least-squares methods using PRISM 3 and the Cheng–Prusoff equation, as reported earlier^32^^,^^33^ and represent the mean from at least three different determinations. All CA isoforms were recombinant ones obtained in-house as reported earlier^34–38^.

### *2.4.* In vitro *anti-proliferative activity*

Ten of the newly synthesised 1,2,3-triazolo benzenesulfonamide derivatives were selected by NCI (Bethesda, MD) for evaluating their anti-proliferative activity. The selected compounds were subjected to a primary *in vitro* one-dose (10 mM) anti-proliferative assay against 57 human tumour cell lines following the previously reported method^30^ and their obtained growth inhibition percent (GI%) are presented in Table 2.

**Table 2.** Percentage growth inhibition (GI%) of *in vitro* human tumour cell lines at 10 µM concentration for ten compounds.

Panel/Cell line	Compound									
	3	5	7	9	11	13	16	17	18	20
*Leukaemia*										
CCRF-CEM	15.3	11.3	16.4	15.9	6.7	13.8	16.4	11.4	17.8	20.9
HL-60(TB)	6.5	22.5	11.6	12.6	10.7	15.9	5.5	12.5	26.1	8.9
MOLT-4	13.3	7.7	10.2	6.9	11.2	8.4	17.6	2.3	21.3	9.1
RPMI-8226	8.5	8.7	12.5	12.3	7.1	7.5	8.4	12.5	12.3	12.1
SR	3.5	4.1	9.3	15.8	9.2	6.1	16.8	3.2	1.7	7.5
*Non-small cell lung cancer*										
A549/ATCC	4.2	7.2	6.7	16.1	11.8	7.7	8.6	16.3	5.1	12.2
EKVX	8.7	8.6	9.2	4.2	2.0	8.3	39.5	17.2	3.7	3.1
HOP-62	8.0	7.9	8.3	0.1	4.2	4.2	7.3	21.7	4.8	–
HOP-92	8.2	3.9	13.5	12.8	4.2	12.5	35.4	18.5	10.6	18.0
NCI-H226	5.2	4.6	11.8	4.8	–	4.2	9.8	16.9	9.8	6.9
NCI-H23	–	–	2.1	–	4.1	5.9	11.2	9.8	4.7	9.4
NCI-H322M	0	6.6	8.8	–	2.6	–	–	3.9	2.2	3.2
NCI-H460	–	–	–	–	–	–	–	0.9	–	–
NCI-H522	9.7	5.1	16.1	10.5	10.3	3.6	9.8	14.8	13.7	7.8
*Colon cancer*										
COLO 205	5.1	–	–	–	–	–	–	17.0	–	–
HCC-2998	–	–	–	–	–	3.3	–	3.9	–	2.2
HCT-116	7.1	5.4	5.1	1.2	2.1	2.2	4.6	3.6	–	–
HCT-15	–	4.1	1.7	4.3	–	1.9	1.6	1.2	–	–
HT29	9.4	–	4.5	0.4	5.1	–	1.2	3.3	10.8	–
KM12	–	5.2	0.6	0.5	–	2.6	–	5.9	–	–
SW-620	5.1	–	2.8	–	2.3	0.9	–	2.7	4.3	–
*CNS cancer*										
SF-268	2.8	2.5	6.5	–	–	–	5.4	3.9	1.5	–
SF-295	–	1.8	–	0.3	–	0.7	5.7	3.1	–	–
SF-539	1.9	1.3	–	–	3.2	4.8	–	2.7	–	–
SNB-19	2.9	3.8	7.8	–	2.8	0.9	3.4	0.8	1.7	1.9
SNB-75	15.0	14.5	20.1	2.9	10.2	13.3	20.0	21.6	13.9	3.3
U251	0.6	7.7	6.4	4.7	10.9	2.8	2.7	12.6	–	1.9
*Melanoma*										
LOX IMVI	6.1	5.5	5.9	–	–	5.5	20.4	9.6	7.5	4.6
MALME-3M	–	–	3.6	–	1.2	2.7	–	0.4	–	–
M14	2.7	4.7	2.6	–	3.3	1.6	–	3.5	–	–
MDA-MB-435	3.1	0.4	8.3	3.4	–	–	2.9	–	–	–
SK-MEL-2	–	–	2.2	–	–	–	–	2.3	–	8.2
SK-MEL-28	–	–	–	–	–	–	–	–	–	0
UACC-257	10.2	11.1	11.7	2.3	8.1	8.6	6.9	8.4	6.1	6.2
UACC-62	5.5	3.4	4.0	6.0	5.9	8.4	15.5	11.6	5.6	4.3
*Ovarian cancer*										
IGROV1	–	–	0.5	–	3.3	–	16.4	12.1	–	–
OVCAR-3	–	–	–	–	–	–	–	–	–	–
OVCAR-4	4.1	–	10.2	–	–	–	21.6	0.8	7.9	–
OVCAR-5	–	3.4	–	–	–	–	–	3.4	–	–
OVCAR-8	4.6	–	2.4	7.4	2.9	4.1	3.8	11.4	3.7	4.8
NCI/ADR-RES	–	–	–	5.4	3.2	0.3	4.2	–	–	2.2
SK-OV-3	4.6	3.5	8.2	9.9	15.1	6.7	9.7	12.3	11.6	3.6
*Renal cancer*										
786-0	–	2.1	–	6.1	–	1.1	–	–	–	–
A498	13.6	8.6	–	8.6	–	0.8	17.6	1.8	5.5	6.2
ACHN	1.3	–	0.6	–	–	–	–	9.3	–	–
RXF 393	–	–	–	–	–	–	17.8	–	–	–
SN12C	1.1	11.8	2.9	–	1.6	2.3	8.9	4.7	1.2	0.9
TK-10	9.2	3.6	–	7.6	–	–	–	–	–	–
UO-31	14.5	13.5	21.1	0.3	13.4	12.7	25.0	29.9	13.8	7.3
*Prostate cancer*										
PC-3	8.3	10.3	15.9	9.3	9.7	13.0	22.2	13.8	7.8	9.7
DU-145	–	–	–	1.0	–	–	–	–	–	–
*Breast cancer*										
MCF7	5.5	9.1	6.7	4.7	1.7	1.9	13.6	7.3	2.9	4.9
MDA-MB-231/ATCC	–	–	4.9	–	–	7.4	24.2	18.0	14.8	–
HS 578T	5.9	99.8	1.7	–	–	1.7	12.1	2.9	9.8	2.5
BT-549	–	3.1	2.0	11.3	–	0.1	–	0.3	–	–
T-47D	5.1	4.8	4.6	11.5	9.6	4.8	5.6	5.2	5.8	–
MDA-MB-468	–	1.3	3.3	–	–	–	–	3.2	3.5	–

## 3. Results and discussion

### 3.1. Chemistry

**Scheme 1.**
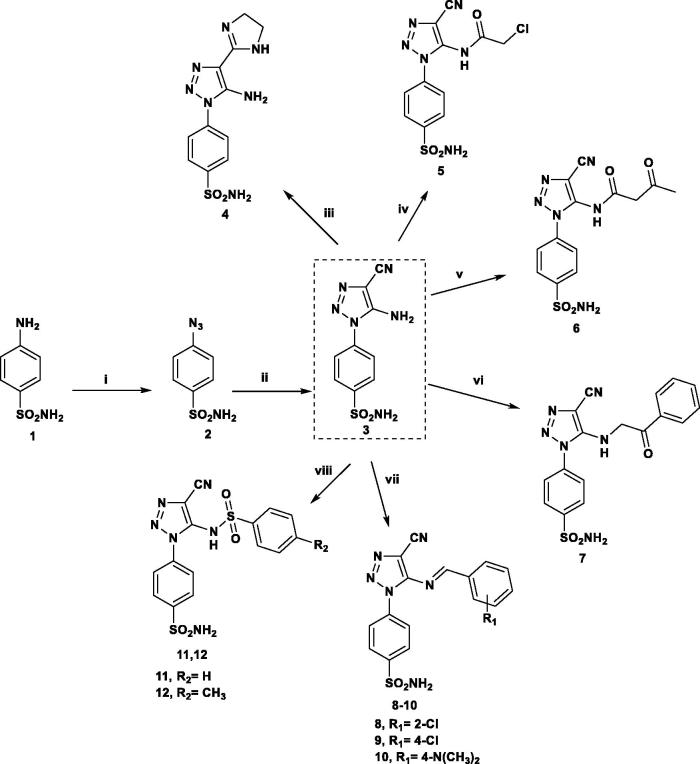
Reagents and conditions: (i) NaN_3_/H_2_SO_4_/NaNO_2_/r.t.; (ii) CH_2_(CN)_2_/EtONa/EtOH/r.t.; (iii) NH_2_CH_2_CH_2_NH_2_/CS_2_/reflux 6 h; (iv) ClCH_2_COCl/DMF/r.t.; (v) CH_3_COCH_2_COOC_2_H_5_/reflux 3 h; (vi) PhCOCH_2_Br/EtOH/reflux 3 h; (vii) Ar-CHO/AcOH/reflux 5 h; (viii) Ar-SO_2_Cl/pyridine/8 h.

Synthesis of the series of 1,2,3-triazolo-benzensulfonamide derivatives **3**–**21** begins with 4-azido benzenesulfonamide^32^ a which was subjected to reaction with malononitrile under stirring at room temperature to give *4-(5-amino-4-cyano-1H-1,2,3-triazol-1-yl)benzenesulfonamide***3**, which represents the key intermediate to produce the target compounds **4**–**21**. Reaction of **3** with ethylene diamine and carbon disulfide as catalyst^32^b afforded the imidazoline derivative **4** whose structure was confirmed by disappearance of the carbonotrile band in IR and the presence of the corresponding protons and carbons of imidazoline ring in NMR spectra. Substitution on the amino group of **3** proceeded successfully via simple reactions with chloroacetyl chloride, ethyl acetoacetate and phenacyl bromide affording the corresponding compounds **5**–**7** in good yields. The Schiff’s bases **8**–**10** were obtained by reaction of **3** with substituted aromatic aldehydes, and in compounds **11** and **12**, a new sulfonamide moiety is introduced to the amino group of **3** through reaction with benzene/toluene sulfonyl chloride (Scheme 1). In compounds **5**–**12** the ^1^H-NMR spectra showed the disappearance of NH_2_ signals and the presence of the corresponding signals for the introduced groups as listed in the experimental section.

**Scheme 2.**
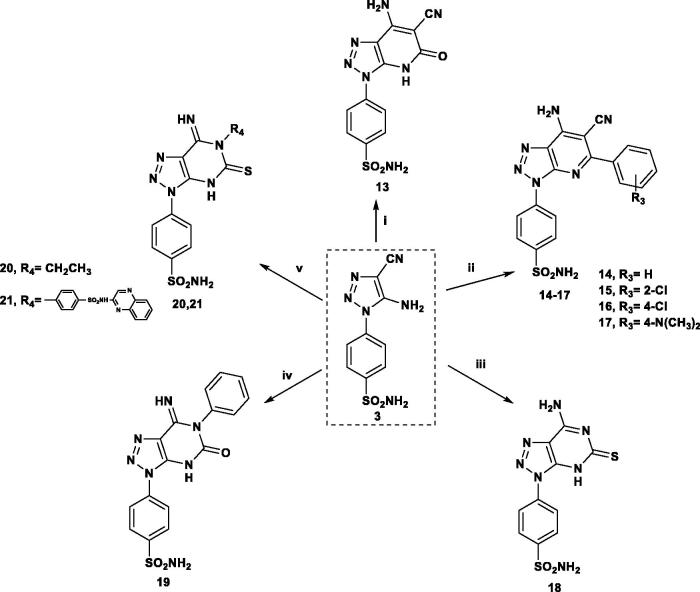
Reagents and conditions: (i) CNCH_2_COOC_2_H_5_/3 h; (ii) Ar-CHC(CN)_2_/EtOH/TEA/5 h; (iii) NH_2_CSNH_2_/15 min; (iv) PhNCO/DMF/TEA/reflux 18 h; (v) CH_3_CH_2_ or Ar-NCS/DMF/TEA/reflux 5 h.

The triazole derivative **3,** bearing two active functional groups, was further cyclised by reaction with 3-ethylcyanoacetate and different 4-substituted benzylidene derivatives affording the corresponding triazolo-pyridine derivatives **13**–**17**. On the other hand, reaction of **3** with thiourea and phenyl thiocyanate afforded the triazolo-pyrimidines **18** and **19**. Similarly, the isothiocyanate derivatives reacted with **3** to give the triazolo-pyrimidine derivatives **20** and **21** bearing ethyl or sulfaquinoxaline moiety, respectively (Scheme 2).

The structures of the target compounds were proved by elemental and spectral data and were in consistency with assigned structures as presented in details in the experimental section.

### 3.2. Carbonic anhydrase inhibition

The synthesised compounds were tested against the cytosolic hCA I, II the transmembrane IV and the tumour-associated membrane bound hCAIX by a stopped-flow CO_2_ hydrase assay in comparison to acetazolamide (AAZ) as standard CAI^31^. The results presented in Table 1 allowed to depict the following SAR.

**Table 1.** Inhibition data of human CA isoforms hCA I, II, IV and IX with compounds 2–21 reported here and the standard sulfonamide inhibitor acetazolamide (AAZ) by a stopped flow CO_2_ hydrase assay.

Compound	K_I_ (nM)			
	hCA I	hCA II	hCA IV	hCA IX
**2**	1267.5	700.4	>10000	235.5
**3**	1940.7	16.7	2272.8	117.7
**4**	9938.3	6836.6	3186.2	3707.4
**5**	2545.5	518.1	1199.9	454.1
**6**	2855.9	683.9	471.7	410.1
**7**	1789.1	562.3	744.7	283.0
**8**	5139.6	6127.7	>10000	1772.5
**9**	1601.2	94.5	4667.3	306.8
**10**	2922.9	178.7	3414.4	451.0
**11**	5549.1	356.4	4522.1	144.0
**12**	8101.8	90.1	2606.9	105.3
**13**	789.7	957.0	166.8	286.7
**14**	1247.8	57.0	675.5	46.4
**15**	2690.5	230.2	2577.4	407.1
**16**	604.8	168.6	788.1	42.6
**17**	682.6	56.8	473.3	35.1
**18**	955.4	732.3	489.1	272.3
**19**	2800.5	589.1	2270.2	158.2
**20**	908.5	929.5	874.6	241.8
**21**	5835.2	2374.7	5030.0	3809.6
**AAZ**	250	12	74	25

The cytosolic hCA I was moderately inhibited by all the tested compounds with inhibition constant (K_I_) ranging from 604.8 to 9938.3 nM. The most active compounds were the triazolo-pyridine derivatives **13**, **16** and **17** (789.7, 604.8 and 682.6 nM, respectively).

The physiologically dominant isoform hCA II was moderately inhibited by all the synthesised compounds with different extents having inhibitory constants in the range of 16.7–6836.6 nM The *4-(5-amino-4-cyano-1H-1,2,3-triazol-1-yl)benzenesulfonamide***3** showed the best activity (16.7 nM), very close to that of acetazolamide (12 nM). Moreover, a good activity was observed for the triazolo-pyridine **17** (56.8 nM).

A relatively poor affinity for the tested compounds was observed against hCA IV, with their inhibitory constant spanning between 116.8 and 5030.30 nM, whereas compounds **2** and **8** showed no activity.

The tumour-associated hCA IX was the most significantly inhibited by the tested compounds which showed inhibitory constants ranging from 35.1 to 3809.6 nM. The most active compounds were the triazolo-pyridine derivatives **14**, **16** and **17** (46.4, 42.6 and 35.1 nM, respectively).

Hence, *p*-substitution on benzenesulfonamide with triazolo-pyridine moiety was the most successful towards CA inhibition. Introduction of aryl group to position 5 of triazolo-pyridine led to high affinity towards hCA IX, whereas *p*-substitution on 5-aryl group increased activity especially for the *N*-dimethyl derivative **17,** which is the most potent candidate in this study.

### *3.3.* In vitro *anti-proliferative activity*

A subset of ten triazolo-benzenesulfonamides (**3**, **5**, **6**, **7**, **9**, **11**, **16**–**18**, and **20)** were selected and subjected to an *in vitro* anti-proliferative screening against a panel of 57 cancer cell lines at NCI at an initial high dose (10 mM). The human cell lines used were derived from nine cancer types: leukaemia, lung, colon, CNS, melanoma, ovarian, renal, prostate and breast cancers. The mean percentages growth inhibition (GI%) of the tested compounds are shown in Table 2.

The tested compounds showed fair anti-proliferative activity over the different cell lines. They inhibited cell growth by different extents and their sensitivity varies as presented in the table. The tested compounds were active mostly against leukaemia and lung cancer cell lines. Compound **16** was sensitive against 9 cell lines showing the highest GI% on EKVX (39.5%) and HOP-92 (34.4%) lung cancer cell lines. Compound **17** was active against 12 cell lines with the highest GI% on SNB-75 CNS cancer cell line (21.6%). Compound **18** was mostly active on HL-60(TB) Leukaemia cell line with GI% = 26.1%. While, compound **20** showed highest activity (20.9%) on CCRF-CEM leukaemia cell line. An exceptional activity was observed for compound **5** on HS 578 T breast cancer cell line showing GI% of 99.8%.

## 4. Conclusions

The present work describes the design and synthesis of novel series of 1.2.3-triazolo benzensulfonamide derivatives according to the general pharmacophoric requirements of the tumour-associated hCA IX. The synthesised compounds were tested *in vitro* against four human carbonic anhydrase isozymes (hCA I, hCA II, hCA IV and hCA IX). hCA IX was the most sensitive isozyme towards the tested compounds, with the most active inhibitors being the triazolo-pyridine benzenesulfonamides **14**, **16**, and **17**. In addition, they showed fair anti-proliferative activity against a panel of 57 human tumour cell lines. These results present a novel group of selective hCA IX inhibitors having triazolo-pyridine benzensulfonamide moiety. Future work will focus on varying hydrophobic substitutions on the pyridine ring to enhance selectivity.
